# Ailanthone Inhibits Cell Proliferation in Tongue Squamous Cell Carcinoma via PI3K/AKT Pathway

**DOI:** 10.1155/2022/3859489

**Published:** 2022-11-01

**Authors:** Shuhan Wang, Qixiao Cui, Xiaoyu Chen, Xuejie Zhu, Kehao Lin, Qiusheng Zheng, Yuliang Wang, Defang Li

**Affiliations:** ^1^Collaborative Innovation Platform for Modernization and Industrialization of Regional Characteristic Traditional Chinese Medicine, School of Integrated Traditional Chinese and Western Medicine, Binzhou Medical University, Yantai 264003, Shandong, China; ^2^College of Stomatology, Binzhou Medical University, Yantai 264003, Shandong, China; ^3^College of Stomatology, Qilu Medical University, Zibo 255300, Shandong, China; ^4^Department of Oral and Maxillofacial Surgery, Yantai Affiliated Hospital of Binzhou Medical University, Yantai 264100, Shandong, China

## Abstract

Tongue squamous cell carcinoma (TSCC) is the most widespread and invasive subtype of oral cancer with high recurrence rates. Ailanthone (AIL) is an active ingredient in the plant extracts of *Ailanthus altissima (Mill*.*) Swingle*. Here, we showed that AIL inhibited the proliferation of human TSCC, the cell viability of Cal-27 and Tca8113 was significantly decreased after AIL treatment for 24 h. Hoechst 33258 staining demonstrated apoptotic characteristics (such as chromatin aggregation) after AIL treatment. The ratio of early- and late-apoptotic cells in AIL-treated Cal-27 and TCA8113 cells increased remarkably when compared with the control group. Bcl-2/Bax ratio and the levels of PARP1, caspase-9, and caspase-3 decreased after AIL treatment, accompanied by significant increase of cleaved PARP1, cleaved caspase-9, and caspase-3 in Cal-27 and TCA8113 cells. Meanwhile, AIL led to Cal-27 cell cycle arrest at G2/M phase. Western blot implied decreased levels of CDK1 and cyclin B1 after AIL treatment. The level of phospho-PI3K p55 subunit and p-Akt were significantly downregulated by AIL in both Cal-27 and TCA8113 cells. These findings implied the potential applications of AIL in the treatment of human TSCC.

## 1. Introduction

TSCC is the most widespread and highly invasive oral squamous carcinoma [1]. TSCC can easily lead to disorders of speech, swallowing, and chewing, thereby seriously restricting the patients' quality of life [2]. It also has a poor prognosis and a high local recurrence rate, which lead to reduced overall survival rate. In recent years, death rate caused by TSCC has significantly increased, and the incidence of TSCC has shifted to a younger age [3]. However, the surgical therapy frequently led to maxillofacial deformity, speech disorder, eating difficult, and other functional disorders, with a high rate of relapse and lymphatic metastasis or distant tissue metastasis [4]. Therefore, it is urgent to explore novel drugs or targets to treat TSCC.

Recent research has focus on the antitumor efficacies of traditional Chinese medicines (TCM) based on their verified pharmacological effects and fewer adverse reactions. TCM exert their therapeutic effect in a comprehensive way to the whole-body system by maintaining a normal balance. Moreover, the specialists of traditional Chinese medicines tend to elevate the body's endogenous resistance to disease and individualize treatment. Naturopathic therapy with TCM is chosen by a high number of patients, considering that these substances are considered as multicomponent, multitarget, and multistage agents. TCM approach is being accepted by more and more people all over the world.

Ailanthone (AIL) is an active ingredient in the plant extracts of traditional Chinese medicine *Ailanthus altissima (Mill*.*) Swingle* [5], which has been employed to treat ascariasis, diarrhea, gastrointestinal diseases, bleeding, and inflammation [6, 7]. Moreover, AIL has been reported to exert growth inhibition in several cancer *in vitro* and *in vivo* [8]. Ni et al. found that AIL inhibited cell proliferation in nonsmall cell lung cancer (NSCLC) and suppressed tumor growth in xenografted and orthotopic lung cancer models, which led to prolonged survival of the two types of tumor-bearing mice [9]. AIL also induced cell cycle arrest at G0/G1 phase, as indicated by up-regulated levels of p21 and p27 and down-regulated levels of cyclins and CDKs in hepatocellular carcinoma (HCC) [10].

To date, there have been no studies about the effect and mechanism of AIL on human TSCC. Therefore, this study used tongue squamous carcinoma Cal-27 and Tca8113 cells to evaluate the antitumor effect of AIL and tried to clarify its potential mechanism.

## 2. Materials and Methods

### 2.1. Reagents and Chemicals

Ailanthone (AIL) (purity ≥ 98%) was ordered from Jiangxi Herb Tiangong Technology (Jiangxi, China). The acquisition of Dulbecco's Modified Eagle's Medium (DMEM) and RPMI-1640 medium was from HyClone (Logan, UT). Fetal bovine serum (FBS) was acquired from Thermo Fisher Scientific (Shanghai, China). In addition, phenylmethylsulfonyl fluoride (PMSF), 3-(4,5-dimethylthiazol-2-yl)-2,5-diphenyltetrazolium-bromide (MTT), and RIPA lysis buffer were provided by Sigma-Aldrich (Stein Heim, Germany). The possession of Penicillin/streptomycin (1 : 100), Hoechst 33258 dye, protease and phosphatase inhibitors, cell-cycle detection kit (CCDK), and Annexin V-FITC/PI Apoptosis Detection Kit (ADK) were acquired from a Chinese company (i.e., Solarbio, Inc, Beijing). BCA-protein assay kits were provided by Nanjing KeyGen Biotech Co., Ltd. (China). Cleared-PARP1 (ab32064, 1 : 5000), PARP1 (ab191217, 1 : 500), cleared caspase-3 (ab32042, 1 : 1000), and cleared caspase-9 (ab2324, 1 : 500) antibodies were ordered from Abcam. Bcl-2 (cat. no. 4223S, 1 : 1000), Bax (cat. no. 5023S, 1 : 1000), caspase-9 (cat. no. 9502S, 1 : 1000), caspase-3 (cat. no. 14220S, 1 : 1000), p-PI3K (cat. no. 17366, 1 : 1000), PI3K (cat. no. 60225-1, 1 : 1000), p-AKT (cat. no. 4060, 1 : 1000), AKT (cat. no. 9272, 1 : 1000), and *β*-actin (cat. no. 4970S, 1 : 1000) antibodies were obtained from CST (Shanghai, China), while horseradish peroxidase-conjugated antirabbit immunoglobulin G (cat. no. D110065) was ordered from Sangon Biotech Co., Ltd (Shanghai, China).

#### 2.1.1. Cell Culture

Human tongue squamous cell carcinoma Cal-27 and Tca8113 cells were purchased from Yantai Bayu Biotechnology Co. Ltd. The Cal-27 cells were grown on 89% DMEM with 1% penicillin/streptomycin and 10% FBS. The Tca8113 cells were grown on RPMI-1640 medium containing 10% FBS and 1% penicillin/streptomycin. The cells were incubated in a sterile incubator (HF240, HEALFORCE, Shanghai, China). After the cells had grown to about 80% of the culture flask, passaging was carried out, and the passage was carried out once in a ratio of 3 : 1 in about 2-3 days.

#### 2.1.2. MTT Assay

Cal-27 and Tca8113 cells at a density of 5 × 10^3^ were seeded into 96-well plates and kept at 37°C and 5% CO_2_. The cells were incubated with different concentrations of AIL (0.25, 0.5, 1, 2, 4, 8, 16, and 32 *μ*M) for 24 h. Then, 10 *μ*L MTT (Sigma-Aldrich, Germany) PBS solution was added and subsequently incubated for 4 h. After incubation, the formazan crystals were dissolved with 200 *μ*L DMSO. The optical density (OD) of each well was measured at 490 nm using a microplate reader (Tecan Group, Switzerland). All OD values were normalized to the control group cells.

#### 2.1.3. Hoechst 33258 Staining

Morphological changes in cell nuclei were measured using Hoechst 33258 probe, as described previously [11]. Cal-27 and Tca8113 cells at a density of 5 × 10^4^ cells/mL were seeded onto 24-well plates and cultured for 24 h. The cells were incubated with different concentrations (0.25, 1, and 4 *μ*M) of AIL for 24 h. After the incubation, the cells were fixed with 4% paraformaldehyde at room temperature for 10 min. After rinse with PBS, the cells were incubated with Hoechst 33258 staining solution in the dark for 15 min. After the cells were washed thrice with PBS, the pictures of the stained nuclei were observed and obtained by a fluorescence microscope (DMI3000B, Leica, Germany).

#### 2.1.4. Evaluation of Apoptosis

Briefly, 2 × 10^5^ Cal-27 and Tca8113 cells were seeded onto 6-well plates and kept at 37°C and 5% CO_2_. After 24 h treatment with AIL (0.25, 1, and 4 *μ*M), the cells were digested and resuspended in 400 *μ*L of Annexin V binding buffer. Then, 5 *μ*L FITC-conjugated Annexin V solution and 5 *μ*L PI solution were added and incubated for 15 min in the dark at room temperature. Finally, the cells were counted and analyzed by FACScan flow cytometer and BD FACSuite™ software.

#### 2.1.5. Analysis of Cell Cycle

Logarithmic growth phase of cells (2 × 10^5^) i.e., Cal-27 and TCA8113 cells were inoculated on 6-well plates. The second day, when the cell density reached about 70%, different concentrations of AIL were added, followed by overnight incubation. The 6-well plates were taken out and the cells were collected. The precooled 75% alcohol was separately added and placed at 4°C for 24 hrs. On the next day, the cells were cleaned with cold PBS, and centrifuged, followed by addition of 100 *μ*L RNase A (Cat. No. CA1050, Solarbio, China), and then incubation at 37°C for 30 min, and addition of 400 *μ*L PI staining solution. Next, the cells were incubated in dark for 0.5 h (at 4°C). Finally, the cell cycle distribution was analyzed by flow cytometry.

#### 2.1.6. Western Blotting

Logarithmic growth phase of 2 × 10^6^ cells i.e., Cal-27 and TCA8113 were inoculated on Petri dishes (100-mm). Next, either vehicle or AIL treated the cells. Then, the cells washing (twice) were carried out with cold PBS, followed by solubilizing in lysis buffer with phosphatase and protease inhibitors. The BCA protein detection kit was used for protein concentration. The protein separation using a total of 40 *μ*g cell lysate were used by 10% SDS-PAGE, followed by protein transferring to PVDF membranes (EMD Millipore). In 2 hours, PVDF membranes were blocked with 5% nonfat milk under the condition of room temperature. Then, the PVDF membrane diluted in 5% nonfat milk in TBST with Tween 20 (0.1%) was incubated with special antibodies or anti-*β*-actin as housekeeping protein. Subsequently, horseradish peroxidase-conjugated IgG served as secondary antibody were used for the incubation. The detection of secondary antibodies on the PVDF membrane were used by the enhanced chemiluminescence (ECL) detection reagents (Pierce, Thermo Fisher). Densitometry analysis (including integrated density of bands) was carried out via Image J (NIH), followed by normalizing the documented values to beta-actin.

### 2.2. Statistical Analysis

Each experiment was carried out at least three times and the data were supplied as mean ± Standard Error of Mean (SEM). An unpaired Student's *t*-tests or one-way ANOVA followed by Tukey's post hoc test was performed using SPSS 25.0 statistical software. *P* < 0.05 was considered statistically significant.

## 3. Results

### 3.1. AIL Suppresses Cell Activities and Induces Apoptosis of TSCC Cells

**Figure 1:**
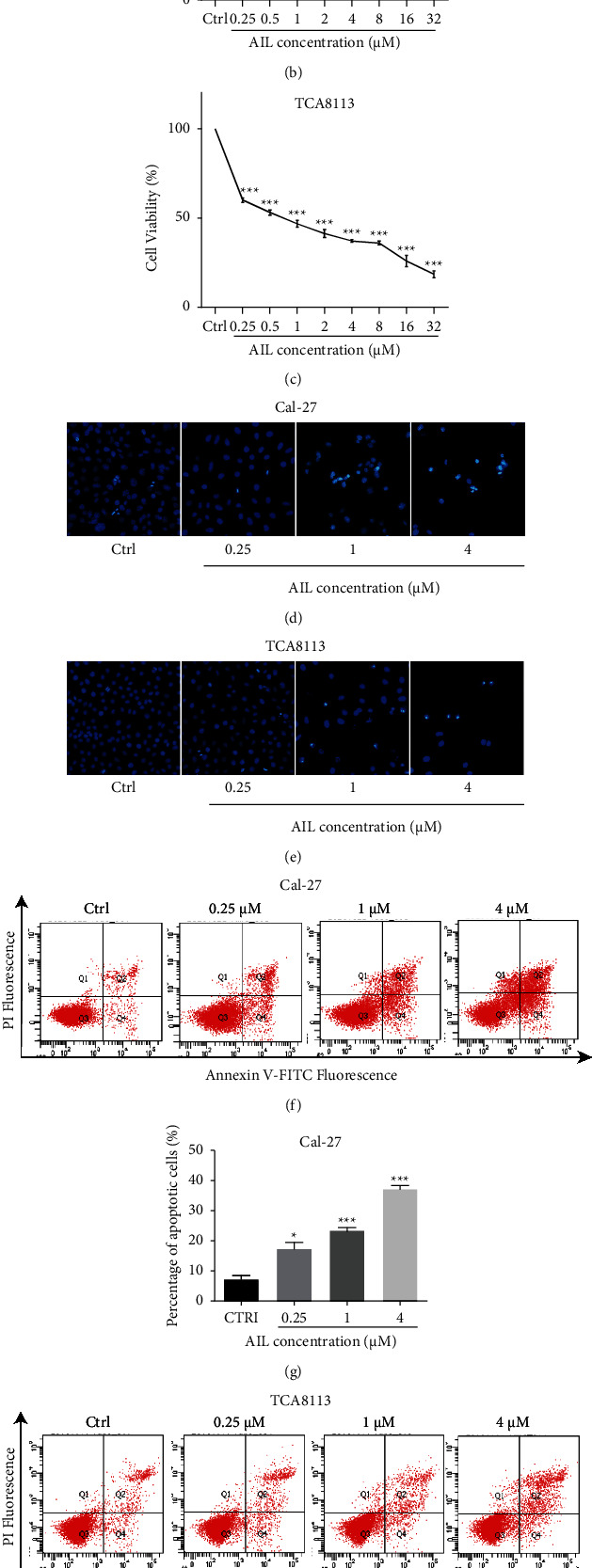
AIL reduces cell activity and induces cell apoptosis in TSCC cells. (a) Chemical structure of AIL. The cell activities of Cal-27 (b) or TCA8113 (c) cells was examined after treatment with AIL for 24 h ^*∗∗∗*^*P* < 0.001 versus the control. Influence of AIL on apoptosis among Cal-27 (d) or TCA8113 (e) cells, as detected by Hoechst 33258 staining. The apoptotic rate of Cal-27 (f-g) or TCA8113 (h-i) cells after AIL treatment were detected and analyzed using flow cytometry. ^*∗*^*P* < 0.05, ^*∗∗*^*P* < 0.01, and ^*∗∗∗*^*P* < 0.001, in comparison with the control cells.

A range of concentrations (0.25, 0.5, 1, 2, 4, 8, 16, and 32 *μ*m) of AIL (Figure 1(a)) was employed in an MTT assay to detect the cell activity for 24 h. The data showed that that the viability of Cal-27 and TCA8113 cells was significantly reduced (The IC_50_ values corresponding to Cal-27 cells and TCA8113 cells were 0.8408 and 0.7884, respectively) (Figures 1(b) and 1(c)). With different concentrations of AIL, the number of TCA8113 and Cal-27 cells decreased under an inverted fluorescence microscope. Hoechst 33258 staining revealed that the shape of nuclei shrank in TCA8113 and Cal-27 cells after the exposure to AIL. Following 1 *μ*M and 4 *μ*M AIL treatment, the signal of chromatin aggregation was observed in TCA8113 cells and Cal-27 (Figures 1(d) and 1(e)). Flow cytometry was further used to detect apoptosis by using double staining based on Annexin V-FITC/PI. When juxtaposed against the control group, among the TCA8113 and Cal-27 cells, the number of cells in the early and late stages of apoptosis manifested an increase (Figures 1(f) and 1(h)). As shown in Figures 1(g) and 1(i), apoptotic cells had a percent count of 17% in TCA8113 cells and 39% in Cal-27 after treatment with 4 *μ*M AIL.

### 3.1.1. AIL Induces Mitochondrial-Mediated Apoptosis in TSCC Cells

**Figure 2:**
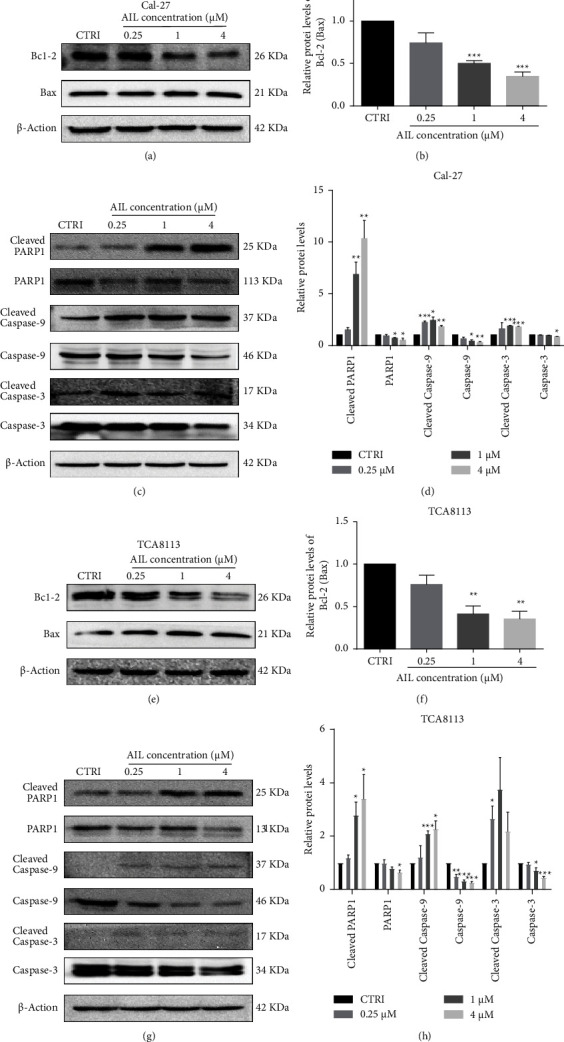
AIL induced apoptosis in TSCC by influencing Bcl-2/Bax ratio and caspase expression. (a) The levels of Bax and Bcl-2 in Cal-27 cells subjected to AIL treatment were assessed by western blotting. (b) Statistical study of the Bcl-2/Bax ratio in Cal-27 cells with or without AIL. ^*∗∗∗*^*P* < 0.001 versus the control. (c-d) Western blotting analysis of the levels of PARP1, caspase-3 and -9, cleaved PARP1, and cleaved caspase-3 and -9 in AIL-treated Cal-27 cells. ^*∗*^*P* < 0.05, ^*∗∗*^*P* < 0.01, and ^*∗∗∗*^*P* < 0.001 versus the control. (e-f) Western blotting analysis of the protein expression of Bcl-2 and Bax in TCA8113 cells treated with or without AIL. ^*∗∗*^*P* < 0.01 versus the control. (g-h) Western blotting analysis of the levels of PARP1, caspase-3 and -9, cleaved PARP1, and cleaved caspase-3 and -9 in AIL-treated TCA8113 cells. ^*∗*^*P* < 0.05, ^*∗∗*^*P* < 0.01, and ^*∗∗∗*^*P* < 0.001 versus the control.

Previous data demonstrated that AIL treatment of TCA8113 and Cal-27 cells subsequently resulted in an increment in the count of apoptotic cells in the early and late stages. To further confirm that AIL induces Cal-27 and TCA8113 cell apoptosis, we employed western blot for detecting the expression of proteins associated with apoptosis. We found that Bcl-2/Bax ratio was significantly decreased after 1 *μ*M AIL and 4 *μ*M AIL in both TCA8113 and Cal-27 cells in comparison with the control group (Figures 2(a), 2(b), 2(e), and 2(f)). We further found that following AIL exposures, the expression of PARP1, caspase-9 and -3 underwent a decrease, simultaneously accompanied by cleaved PARP1, cleaved caspase-9 and -3 accumulating in significant quantities within TCA8113 and Cal-27 cells (Figures 2(c), 2(d), 2(g), and 2(h)). The results implies that the inherent apoptotic pathway was primarily responsible for apoptosis induced by AIL in TCA8113 and Cal-27 cells.

### 3.1.2. AIL Triggers the Cell Cycle Arrest at G2/M Phase in Cal-27 Cells but Does Not Influence TCA8113 Cells

**Figure 3:**
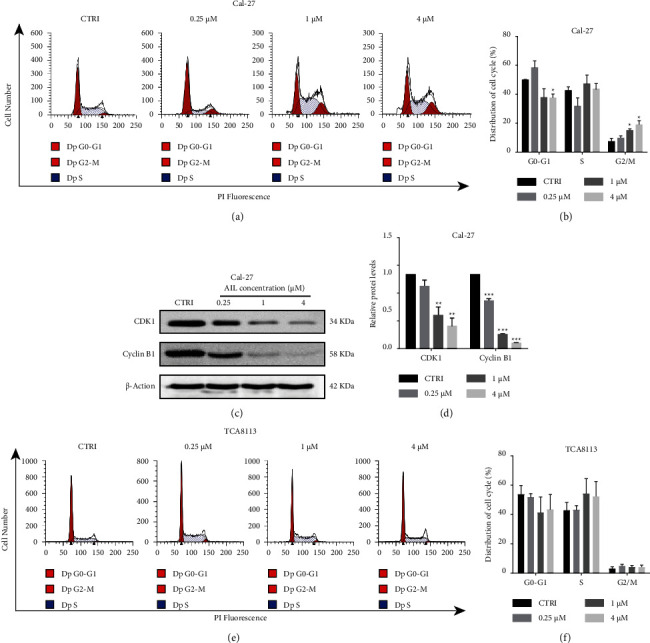
AIL induced cycle arrest in G2/M phase in Cal-27 cells, however, did not have any influence on TCA8113 cells. (a-b) Flow cytometry analysis of the cell cycle distribution changes in Cal-27 cells subjected to AIL treatment or otherwise. ^*∗*^*P* < 0.05, ^*∗∗∗*^*P* < 0.001, compared with the control. (c-d) Western blotting analysis of the levels of CDK1 and Cyclin B1 in Cal-27 cells treated with or without AIL. ^*∗∗*^*P* < 0.01 and ^*∗∗∗*^*P* < 0.001 in comparison with the control. (e-f) Flow cytometry analysis of the changes in cell cycle distribution in TCA8113 cells subjected to AIL treatment or otherwise.

To ascertain if the cell cycle progression is affected by AIL, we subjected Cal-27 and TCA8113 cells to incubation with various concentrations of AIL for a duration of 24 h, followed by measurement and analysis of cell cycle distribution. It was evident that only Cal-27 cells treated with 4 *μ*M AIL manifested a considerable decrease in the ratio of cells within the G0/G1 state, whereas the number of AIL-treated Cal-27 cells at the G2/M phase exhibited a significant increment upon treating with 1 *μ*M and 4 *μ*M AIL compared with control (Figures 3(a) and 3(b)). Thereafter, we made a thorough assessment of the molecular mechanism regulating cell cycle arrest in AIL-treated Cal-27 cells. Western blotting was employed to estimate the G2/M-related proteins. As evident from Figures 3(c) and 3(d), AIL decreased the level of CDK1 and cyclin B1, which implied that AIL induced the arrest of G2/M in Cal-27 cells. We also analyzed the cell cycle change in TCA8113 cells. The percentage of cells at different cell cycle phases did not differ among the AIL treatments in TCA8113 cells (Figures 3(e) and 3(f)). Therefore, we did not further detect the transition in the levels of proteins taking part in the cell cycle.

### 3.1.3. AIL Causes Blocking of the PI3K/AKT Pathway in TSCC Cells

**Figure 4:**
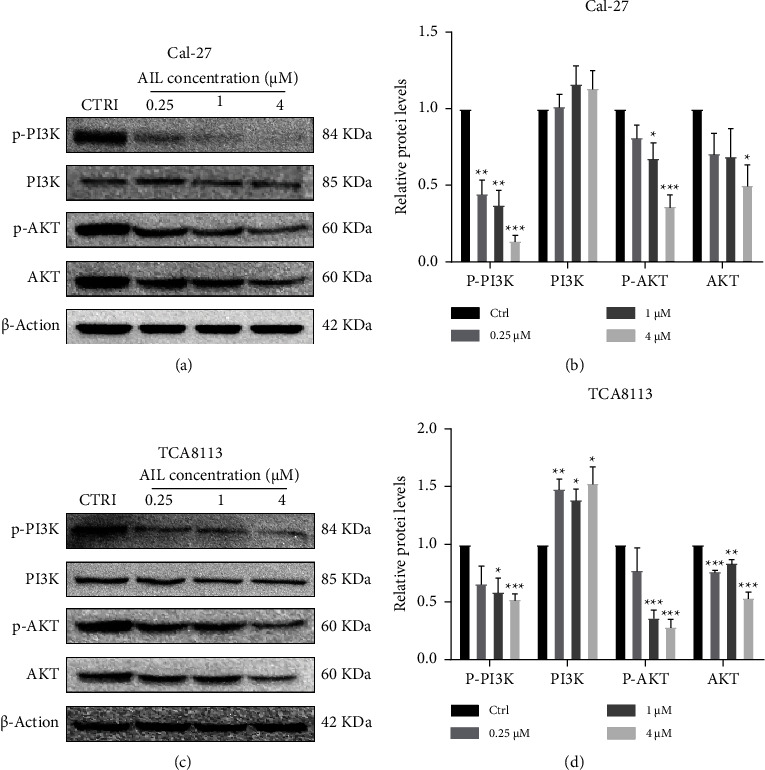
AIL decreased the proliferation of TSCC cells via the PI3K/AKT Pathway. (a-b) Western blotting analysis of the levels of p-PI3K, PI3K, p-AKT, and AKT in the AIL-treated Cal-27 cells. ^*∗*^*P* < 0.05, ^*∗∗*^*P* < 0.01, and ^*∗∗∗*^*P* < 0.001 versus the control. (c-d) The levels of p-PI3K, PI3K, p-AKT, and AKT in TCA8113 cells treated with or without AIL. ^*∗*^*P* < 0.05, ^*∗∗*^*P* < 0.01, and ^*∗∗∗*^*P* < 0.001 versus the control.

To probe into the mechanistic pathway controlling apoptosis and G2/M phase arrest mediated by AIL, an analysis of the levels of PI3K/AKT pathway-associated proteins was carried out. As illustrated in Figures 4(a) and 4(b), the level of phospho-PI3K p55 subunit was significantly downregulated by AIL in Cal-27 cells. As evident in Figure 4(b), the extent of expression of PI3K was not found to significantly alter in Cal-27 cells. From Figures 4(a) and 4(b), the phosphorylation of AKT at the Ser473 site also decreased in Cal-27 cells after AIL treatment. In Cal-27 cells, only after the treatment with 4 *μ*M AIL, the expression of total AKT was significantly decreased (Figure 4(b)). In TCA8113 cells, there was an obvious decrease in the level of phospho-PI3K p55 subunit and phosphorylation of AKT at the Ser473 site by AIL (Figures 4(c) and 4(d)). The level of PI3K was significantly increased after 0.25, 1, and 4 *μ*M AIL in TCA8113 cells (Figure 4(d)), while the level of total AKT was significantly decreased after the treatment with 0.25, 1, and 4 *μ*M AIL (Figure 4(d)).

## 4. Discussion

TSCC is an aggressive form of cancer of the oral cavity and has the highest rate of prevalence and recurrence among the various kinds of oral cancers [1]. In 2015, within China, a total of 48,100 new cases and 22,100 deaths associated with TSCC were reported [12]. Although the clinical outcomes have been improved after the advances in surgical therapy, chemotherapy, and radiotherapy, the prognosis, in general, and the overall survival rates of TSCC patients have not elevated much in the past decade. Hence, it is necessary to explore and find improved drugs for developing novel therapeutic approaches for TSCC patients.

TCM has an extensive background of successful utilization in the treatment and prevention of disorders and diseases. Many studies and clinical observations have revealed that traditional herbal Chinese medicines as well as their extracts have powerful inhibitory characteristics against tumors [13]. AIL, acquired from the Ailanthus altissima, has anticancer potential toward a multitude of cancer cells. In this study, we found that the cell viabilities of TCA8113 and Cal-27 cells were significantly decreased following AIL treatment. Further study showed that exposure to AIL led to chromatin aggregation, and the count of apoptotic cells in the early and late stages of apoptosis among Cal-27 and TCA8113 cells was obviously increased after the treatment with AIL.

It has been reported that the Bcl-2 family proteins play a vital role for cancer cell apoptosis. There are two major types of Bcl-2 family proteins: pro-survival members (e.g., Bcl-2) and pro-apoptotic members (e.g., Bax) [14]. Bcl-2 can be induced by various factors, including growth factor deprivation, glucose deprivation, lipid peroxidation, and many antitumor drugs [15]. The increased level of Bcl-2 has been confirmed to protect against apoptosis [16]. Several clinical studies demonstrated that the higher level of Bcl-2 is related with a poor prognosis in many tumors' types, including pancreatic carcinoma and oral tongue squamous cell carcinoma [17, 18]. Bax, a pro-apoptotic protein, can facilitate mitochondrial membrane permeabilization and activate caspase-3/9, resulting in cell apoptosis [19]. Multiple studies have suggested that decreased Bcl-2/Bax ratio in tumor cells triggers activation of caspase-9/-3 and subsequently promotes the mitochondrial-mediated apoptosis [20, 21]. Consistent with the above results, our data showed that decreased Bcl-2/Bax ratio and increased cleaved caspase-9 and -3 in AIL-treated Cal-27 and TCA8113 cells.

One important characteristic of tumor is uncontrolled cell proliferation, and several clinical drugs exert antitumor effects by suppressing cell proliferation through arresting tumor cell cycle [22]. AIL has been reported that it inhibits Huh7 cell proliferation by blocking cell cycle [10]. In our study, we found that AIL induced cell cycle arrest at G2/M phase in Cal-27 cells, while no significant change of cell cycle distribution in TCA8113 cells after AIL treatment. We supposed that this may be due to the different patients-derived TSCC cells with different cellular metabolic patterns [23]. Cal-27 cells are epithelial cells isolated from a White male with a lesion in the middle of the tongue, and TCA8113 cells are isolated from a biopsy section of tongue carcinoma. Likewise, many studies have showed that the same drug/compound has an obvious different effect in one type of tumor cells from different patients. For example, one study reported that curcumin sensitized TRAIL-induced cytotoxicity in malignant glioma U87MG cells but not in malignant glioma U251MG cells [24]. Several recent studies showed that cyclin d kinase 1 (CDK1) and cyclin B1 are closely related to G2/M cell cycle [25, 26]. Next, we examined the levels of CDK1 and cyclin B1 in AIL-treated Cal-27 cells, and found that the levels of CDK1 and cyclin B1 were remarkably decreased after treatment with AIL. These findings implied that AIL could induce Cal-27 cells G2/M phase arrest through regulation of CDK1 and cyclin B1.

In human cancers, PI3K-AKT pathway is the most recurrently triggered pathway regulating survival, differentiation, cell growth, and cell apoptosis of tumor cells in response to a wide array of signals, and PI3K/AKT inhibitors demonstrate a more effective and also synergize with a variety of chemotherapeutics [27–30]. Phosphorylated AKT promotes the apoptosis-related protein Bcl-2 and cell cycle-related protein cyclin D [31]. The decline in the expression of AKT induces cell apoptosis while inhibiting Bcl-2 expression [32]. It was reported by Zhang et al. that in colorectal cancer, the inhibited phosphorylation of PI3K and AKT reduced Bcl-2 expression subsequently promoting the mitochondrial apoptosis induced by oxaliplatin [33]. In this work, we found that the decreased Bcl-2 expression was accompanied by downregulation of p-PI3K and p-AKT, eventually leading to TCA8113 and Cal-27 cells apoptosis. In addition, according to several reports, the action of the CDK1 and cyclin B1 can be promoted by PI3K/AKT pathway [34, 35]. Our data implied that AIL could downregulate the levels of p-PI3K and p-AKT, and subsequently inhibit the levels of CDK1 and cyclin B1, eventually leading to G2/M cell cycle arrest in Cal-27 cells. Therefore, we can speculate that the downregulation of PI3K/AKT induced by AIL may induce tongue squamous carcinoma cell cycle arrest and apoptosis.

To the best of our awareness, the current work is a pioneer report demonstrating the antitumor effect of AIL on tongue squamous cell carcinoma. For a better understanding of the therapeutic capability of AIL, additional *in vivo* experiments are needed to verify the antitumor ability of AIL. Our observations conclusively demonstrated that AIL instigated cell cycle arrest at G2/M and suppressed the PI3K/AKT pathway to cause apoptosis of tongue squamous cell carcinoma cells. Therefore, this study suggested that AIL showed prospects of being developed into an effective novel therapeutic agent focusing to target the PI3K/AKT pathway in TSCC.

## Data Availability

The data used and/or analyzed during this study are available from the corresponding author upon request.

## References

[1] Gan R. H., Lin L. S., Zheng D. P. (2021). High expression of Notch2 drives tongue squamous cell carcinoma carcinogenesis. *Experimental Cell Research*.

[2] Calabrese L., Pietrobon G., Fazio E. (2020). Anatomically-based transoral surgical approach to early-stage oral tongue squamous cell carcinoma. *Head & Neck*.

[3] Majchrzak E., Szybiak B., Wegner A. (2014). Oral cavity and oropharyngeal squamous cell carcinoma in young adults: a review of the literature. *Radiology and Oncology*.

[4] Zhu L., Wang Y., Li R. (2019). Surgical treatment of early tongue squamous cell carcinoma and patient survival. *Oncology Letters*.

[5] Ding H., Yu X., Hang C. (2020). Ailanthone: a novel potential drug for treating human cancer (review). *Oncology Letters*.

[6] Hou S., Cheng Z., Wang W., Wang X., Wu Y. (2019). Ailanthone exerts an antitumor function on the development of human lung cancer by upregulating microRNA-195. *Journal of Cellular Biochemistry*.

[7] Cucci M. A., Grattarola M., Dianzani C. (2020). Ailanthone increases oxidative stress in CDDP-resistant ovarian and bladder cancer cells by inhibiting of Nrf2 and YAP expression through a post-translational mechanism. *Free Radical Biology and Medicine*.

[8] Peng S., Yi Z., Liu M. (2017). Ailanthone: a new potential drug for castration-resistant prostate cancer. *Chinese Journal of Cancer*.

[9] Ni Z., Yao C., Zhu X. (2017). Ailanthone inhibits non-small cell lung cancer cell growth through repressing DNA replication via downregulating RPA1. *British Journal of Cancer*.

[10] Zhuo Z., Hu J., Yang X. (2015). Ailanthone inhibits Huh7 cancer cell growth via cell cycle arrest and apoptosis in vitro and in vivo. *Scientific Reports*.

[11] Amirbekyan K. Y., Shahinyan G. A., Ghazoyan H. H., Sargsyan H. R., Markarian S. A. (2021). Fluorescence anisotropy studies on the Hoechst 33258-DNA interaction: the solvent effect. *Journal of Biomolecular Structure and Dynamics*.

[12] Chen W., Zheng R., Baade P. D. (2016). Cancer statistics in China, 2015. *CA: A Cancer Journal for Clinicians*.

[13] Tang K. Y., Du S. L., Wang Q. L., Zhang Y. F., Song H. Y. (2020). Traditional Chinese medicine targeting cancer stem cells as an alternative treatment for hepatocellular carcinoma. *Journal of Integrative Medicine*.

[14] Kang Q., Zou H., Yang X. (2018). Characterization and prognostic significance of mortalin, Bcl-2 and Bax in intrahepatic cholangiocarcinoma. *Oncology Letters*.

[15] Singh R., Letai A., Sarosiek K. (2019). Regulation of apoptosis in health and disease: the balancing act of BCL-2 family proteins. *Nature Reviews Molecular Cell Biology*.

[16] Kelly P. N., Strasser A. (2011). The role of Bcl-2 and its pro-survival relatives in tumourigenesis and cancer therapy. *Cell Death & Differentiation*.

[17] Guo Y., Liu H., Chen Y., Yan W. (2020). The effect of allicin on cell proliferation and apoptosis compared to blank control and cis-platinum in oral tongue squamous cell carcinoma. *OncoTargets and Therapy*.

[18] Casneuf V. F., Fonteyne P., Van Damme N. (2008). Expression of SGLT1, Bcl-2 and p53 in primary pancreatic cancer related to survival. *Cancer Investigation*.

[19] Liu Z., Ding Y., Ye N., Wild C., Chen H., Zhou J. (2016). Direct activation of bax protein for cancer therapy. *Medicinal Research Reviews*.

[20] Ma Z. J., Lu L., Yang J. J. (2018). Lariciresinol induces apoptosis in HepG2 cells via mitochondrial-mediated apoptosis pathway. *European Journal of Pharmacology*.

[21] Karmakar I., Haldar S., Chakraborty M., Chaudhury K., Dewanjee S., Haldar P. K. (2016). Regulation of apoptosis through bcl-2/bax proteins expression and DNA damage by zanthoxylum alatum. *Pharmaceutical Biology*.

[22] Liu K., Zheng M., Lu R. (2020). The role of CDC25C in cell cycle regulation and clinical cancer therapy: a systematic review. *Cancer Cell International*.

[23] Lou J. L., Wang Y., Yao C. J. (2013). Role of DNA methylation in cell cycle arrest induced by Cr (VI) in two cell lines. *PLoS One*.

[24] Gao X., Deeb D., Jiang H., Liu Y. B., Dulchavsky S. A., Gautam S. C. (2005). Curcumin differentially sensitizes malignant glioma cells to TRAIL/Apo2L-mediated apoptosis through activation of procaspases and release of cytochrome c from mitochondria. *Journal of Experimental Therapeutics and Oncology*.

[25] Yang Y., Xue K., Li Z. (2018). c-Myc regulates the CDK1/cyclin B1 dependentG2/M cell cycle progression by histone H4 acetylation in raji cells. *International Journal of Molecular Medicine*.

[26] Liu W. T., Chen C., Lu I. C. (2014). MJ-66 induces malignant glioma cells G2/M phase arrest and mitotic catastrophe through regulation of cyclin B1/Cdk1 complex. *Neuropharmacology*.

[27] Cordo V., van der Zwet J. C. G., Cante-Barrett K., Pieters R., Meijerink J. P. P. (2021). T-Cell acute lymphoblastic leukemia: a roadmap to targeted therapies. *Blood Cancer Discovery*.

[28] Chiarini F., Fala F., Tazzari P. L. (2009). Dual inhibition of class IA phosphatidylinositol 3-kinase and mammalian target of rapamycin as a new therapeutic option for T-cell acute lymphoblastic leukemia. *Cancer Research*.

[29] Gazi M., Moharram S. A., Marhall A., Kazi J. U. (2017). The dual specificity PI3K/mTOR inhibitor PKI-587 displays efficacy against T-cell acute lymphoblastic leukemia (T-ALL). *Cancer Letters*.

[30] Wang H., Chan K. Y. Y., Cheng C. K. (2022). Pharmacogenomic profiling of pediatric acute myeloid leukemia to identify therapeutic vulnerabilities and inform functional precision medicine. *Blood Cancer Discovery*.

[31] Liu Z. Y., Zhao L., Song Y. S. (2019). Eya2 is overexpressed in human prostate cancer and regulates docetaxel sensitivity and mitochondrial membrane potential through AKT/Bcl-2 signaling. *BioMed Research International*.

[32] Dai Y., Jin S. G., Li X. P., Wang D. X. (2017). The involvement of Bcl-2 family proteins in AKT-regulated cell survival in cisplatin resistant epithelial ovarian cancer. *Oncotarget*.

[33] Zhang L., Chen H. P., Song Y. Q. (2021). MiR-325 promotes oxaliplatin-induced cytotoxicity against colorectal cancer through the HSPA12B/PI3K/AKT/Bcl-2 pathway. *Digestive Diseases and Sciences*.

[34] Kim S. M., Vetrivel P., Ha S. E., Kim H. H., Kim J. A., Kim G. S. (2020). Apigetrin induces extrinsic apoptosis, autophagy and G2/M phase cell cycle arrest through PI3K/AKT/mTOR pathway in AGS human gastric cancer cell. *The Journal of Nutritional Biochemistry*.

[35] Won Y. S., Seo K. I. (2020). Sanggenol L induces apoptosis and cell cycle arrest via activation of p53 and suppression of PI3K/Akt/mTOR signaling in human prostate cancer cells. *Nutrients*.

[36] wang S., Chen X., Zhu X. Ailanthone decreases cell viability in tongue squamous cell carcinoma via PI3K/AKT pathway. https://www.researchsquare.com/article/rs-911669/v911661.

